# Intrinsic excitability changes induced by acute treatment of hippocampal CA1 pyramidal neurons with exogenous amyloid β peptide

**DOI:** 10.1002/hipo.22403

**Published:** 2015-03-25

**Authors:** Francesco Tamagnini, Sarah Scullion, Jon T. Brown, Andrew D. Randall

**Affiliations:** ^1^ Medical School, University of Exeter, Hatherly Building, Streatham CampusExeterEX4 4PSUnited Kingdom; ^2^ School of Physiology and Pharmacology, University of Bristol, University WalkBristolBS8 1TDUnited Kingdom

**Keywords:** membrane properties, amyloidopathy, hippocampus, hyperexcitability, Alzheimer's disease

## Abstract

Accumulation of beta‐amyloid (Aβ) peptides in the human brain is a canonical pathological hallmark of Alzheimer's disease (AD). Recent work in Aβ‐overexpressing transgenic mice indicates that increased brain Aβ levels can be associated with aberrant epileptiform activity. In line with this, such mice can also exhibit altered intrinsic excitability (IE) of cortical and hippocampal neurons: these observations may relate to the increased prevalence of seizures in AD patients. In this study, we examined what changes in IE are produced in hippocampal CA1 pyramidal cells after 2–5 h treatment with an oligomeric preparation of synthetic human Aβ 1–42 peptide. Whole cell current clamp recordings were compared between Aβ‐(500 n*M*) and vehicle‐(DMSO 0.05%) treated hippocampal slices obtained from mice. The soluble Aβ treatment did not produce alterations in sub‐threshold intrinsic properties, including membrane potential, input resistance, and hyperpolarization activated “sag”. Similarly, no changes were noted in the firing profile evoked by 500 ms square current supra‐threshold stimuli. However, Aβ 500 n*M* treatment resulted in the hyperpolarization of the action potential (AP) threshold. In addition, treatment with Aβ at 500 n*M* depressed the after‐hyperpolarization that followed both a single AP or 50 Hz trains of a number of APs between 5 and 25. These data suggest that acute exposure to soluble Aβ oligomers affects IE properties of CA1 pyramidal neurons differently from outcomes seen in transgenic models of amyloidopathy. However, in both chronic and acute models, the IE changes are toward hyperexcitability, reinforcing the idea that amyloidopathy and increased incidence in seizures might be causally related in AD patients. © 2014 The Authors Hippocampus Published by Wiley Periodicals, Inc.

## INTRODUCTION

Beta‐amyloid peptides (Aβ) are widely believed to play an important role in the pathogenesis of Alzheimer's disease (AD) (Hardy and Higgins, 1992; Pimplikar, 2009). This family of peptides are produced by sequential enzymatic cleavage of the transmembrane protein amyloid precursor protein (APP) by the beta and gamma secretase complexes (Citron, 2004). Mutant forms of human APP, which are more readily processed to Aβ, result in familial early‐onset forms of AD, as do mutations in the presenilins, key components of the gamma secretase complex that is involved in enzymatically liberating Aβ from APP (Chow et al., 2010). Central nervous system (CNS) accumulation of soluble Aβ occurs early in the disease process and it grows as the pathology becomes more advanced and Aβ‐rich amyloid plaques also appear within the neuronal parenchyma (Jacobsen et al., 2006; Naslund et al., 2000).

Numerous neurophysiological studies of transgenic mice which overproduce Aβ have been performed over the last 15 years, in the attempt to understand how AD‐associated Aβ pathology disturbs CNS function (Randall et al., 2010). These studies have frequently concentrated on the analysis of synaptic function and plasticity in hippocampus and cerebral cortex (Fitzjohn et al., 2008; Jacobsen et al., 2006; Tamagnini et al., 2012; Witton et al., 2010). More recent studies, including our own, have looked beyond synaptic function and have started to uncover significant alterations in the intrinsic excitability (IE) of single neurones in transgenic mice that over‐produce Aβ. Such changes to IE have been reported in both excitatory and inhibitory neurones of the hippocampus (Brown et al., 2011; Hazra et al., 2013; Kaczorowski et al., 2011; Kerrigan et al., 2013; Minkeviciene et al., 2009; Wykes et al., 2012), the cortex (Hazra et al., 2013; Verret et al., 2012), and cerebellum (Hoxha et al., 2012). Clearly, such alterations to neuronal IE have to be considered along with changes to synaptic function, if we are to understand the mechanisms through which increased levels of Aβ change the activity of neuronal networks.

Our own work on IE has largely focused on hippocampal CA1 pyramidal cells (CA1‐PC) and to date it has involved investigation of four different transgenic Aβ overproducing mouse lines. We have identified changes to the bursting behavior of CA1‐PC and associated changes to the spike after‐depolarization (ADP) (Brown et al., 2011; Kerrigan et al., 2013). We also found differences in the action potential waveform and demonstrated that these likely arise, at least in part, from alterations to the levels of functional voltage‐gated Na^+^ channels (Brown et al., 2011).

Our work has led to us frequently asked whether the changes in IE we see in Aβ overproducing transgenic mice can also be observed when acutely treating brain slices with an exogenous Aβ preparation. In the work presented in this study, we address this question by measuring the IE properties of CA1 hippocampal neurons in brain slices pre‐treated with 500 n*M* soluble human amyloid beta 1–42 peptides (hAβ 1–42). The concentration used in this study has been frequently used for *in vitro* studies of synaptic function and it has been shown to both impair (Lambert et al., 1998; Lauren et al., 2009; Puzzo et al., 2005; Wang et al., 2004) and to facilitate (Li et al., 2009) hippocampal long‐term potentiation (LTP).

## METHODS

### Experimental Animals

Male C57‐BL6/J mice aged 4–5 weeks were used for all experiments. These animals were group housed and maintained on a standard 12:12 h light/dark cycle with access to food and water *ad libitum*.

### Preparation of Brain Slices

All procedures were performed in accordance with UK Home Office legislation set out in the Animals (Scientific Procedures) Act (1986). The preparation of horizontal ventral hippocampal slices was performed as previously described (Brown et al., 2011; Brown and Randall, 2009). In brief, mice were sacrificed by cervical dislocation and the brain was rapidly removed and transferred to an ice cold (∼4°C), sucrose‐based slicing solution comprising (in m*M*): sucrose, 189; D‐glucose, 10; NaHCO_3_, 26; KCl, 3; MgSO_4_, 5; CaCl_2_, 0.1; NaH_2_PO_4_, 1.25, continuously bubbled with carbogen (95% O_2_, 5% CO_2_ gas mixture). The cerebellum, frontal and dorsal parts were removed with single scalpel cuts. The sample was then mounted on a metal plate on the dorsal side (i.e., ventral side up) and 300 μm thickness horizontal sections were prepared using a Leica VT1200 vibratome. After sectioning, slices were submerged in a storage vessel, which contained our standard artificial cerebrospinal fluid (aCSF) consisting of (in m*M*): NaCl, 124; KCl, 3; NaHCO_3_, 26; CaCl_2_, 2; NaH_2_PO_4_, 1.25; MgSO_4_, 1; d‐glucose, 10, and equilibrated with carbogen. The slices were gradually heated to ∼32–34°C for 30 min, after which they were stored at room temperature for 1 h before being treated with either hAβ 1–42 peptide or vehicle.

### Preparation and Application of hAβ 1–42

A preparation of hAβ 1–42 peptide containing low N oligomeric species was prepared as described elsewhere (Jo et al., 2011). In brief, synthetic hAβ 1–42 was purchased from Tocris and dissolved in 100% HFIP (1,1,1,3,3,3‐hexafluoro‐2‐propanol) to a concentration of 1 mg mL^−1^. This solution was incubated at room temperature (RT) for 1 h, vortexing every 5–10 min. The preparation was then sonicated for 10 min at RT. The HFIP/peptide solution was subsequently dried down under a gentle stream of nitrogen. Following this, the dried peptide was re‐suspended in 100% DMSO to a concentration of 1 m*M* and incubated for 12 min at RT, gently rotating the vial and vortexing every few minutes. This preparation was then pipetted into 10 μL aliquots and stored at −80^o^C. On the day of use, a single aliquot was rapidly thawed and 90 μL of PBS 0.1*M* added before performing 90–120 min of rotation and gentle agitation. Following this, the Aβ preparation was finally diluted 1:2000 into standard aCSF to a final Aβ concentration of 500 n*M* and applied to slices for 2–5 h before electrophysiological recording. Control slices were exposed for a similar period to vehicle only (0.05% DMSO); use of this vehicle control group is particularly important as we have shown that this concentration of DMSO can alone change IE of cortical and hippocampal neurons (Tamagnini et al., 2014). Notably, Aβ was not applied during the recording phase of our experiments, thus any changes observed arise from the prior period of pre‐treatment performed before the tissue was transferred to the electrophysiological recording setup.

The most commonly reported neurophysiological actions of oligomeric Aβ preparations are on long‐lasting, activity‐dependent, forms of synaptic plasticity such as LTP and long‐term depression (Randall et al., 2010). Pre‐incubating cortical slices with the Aβ preparation we use here is able to reliably modify synaptic plasticity, completely abolishing the LTD in perirhinal cortex that can be evoked using a 5 Hz conditioning stimulus (S.S., J.B., and A.R. unpublished observations).

### Electrophysiological Methods

Effects of exogenous Aβ on IE were studied using single cell patch clamp recording from CA1‐PC. The recording methods we used are similar to those we used for our previous studies of intrinsic properties of this cell type in both Aβ‐overproducing transgenic mice and healthy aged animals (Brown et al., 2011; Kerrigan et al., 2013; Randall et al., 2012). In brief, slices were placed into a submersion style recording chamber, which was continuously perfused (∼2 mL min^−1^) with carbogen‐equilibrated aCSF at 33 ± 1°C. CA1 pyramidal neurones in the CA1 subfield of the hippocampus were visually identified using infra‐red (IR) differential interference contrast optics. Pipettes were fabricated from borosilicate glass and were fire polished such that their resistance was 2.5–4.5 MΩ when filled with pipette solution. The pipette solution consisted of (m*M*): K‐gluconate, 135; NaCl, 5; HEPES free acid, 10; EGTA, 0.2; Na‐GTP, 0.3; Mg‐ATP, 4; Biocytin 13.4, Alexafluor 488 hydrazide 0.03–0.06, pH 7.3, 280–290 mOsm.

After forming the giga‐seal and then entering the whole cell configuration in voltage‐clamp mode, the amplifier was immediately switched to bridge‐mode current‐clamp, in which all experiments were performed. The pairing of the pipette solution and the aCSF produces a liquid junction potential error of 15 mV; this was corrected for arithmetically in all data sets. All recordings were made using a MultiClamp 700B amplifier (Molecular Devices, Union City, CA). Recordings were low pass filtered (5–10 kHz) and then digitised (20–100 kHz) with a Digidata 1440 and stored on a personal computer using pClamp10 electrophysiology software.

### *In Vitro* Electrophysiology Protocols and Data Analysis

**Figure 1:**
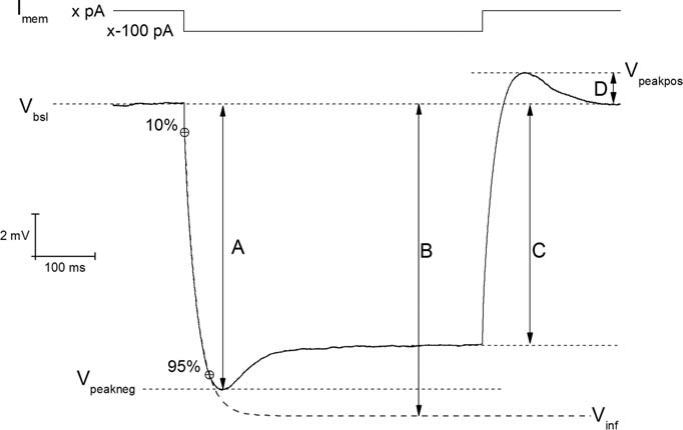
Schematic representation of the determination of passive membrane properties from a hyperpolarizing current step. To assess IE properties of CA1 pyramidal neurons, whole cell current clamp recordings were performed. For each recording, a “holding” current (x pA) was injected to keep the unstimulated *V*_m_ at the fixed value of −80 mV. A 500‐ms long, −100‐pA amplitude current was injected to assess the passive properties of the neuronal membrane. The input resistance was measured at the steady state of the *V*_m_ deflection (arrow C) or at the infinite time extrapolation (arrow B) of the single exponential fit to points between 10–95% of the negative peak (arrow A). The exponential fit was also used to calculate the membrane time constant τ. Sag was calculated both from the decline from the observed negative peak [(A–C)/A)] and from the *V*_m_ extrapolated at the infinite time of the exponential fit [(B–C)/A)]. Sag is mirrored by a positive rebound of the potential following the interruption of the hyperpolarizing current step (D).

Analysis of current‐clamp recordings, including action potential waveform analysis was performed with custom‐written routines within the Matlab environment. Resting potential was measured as soon as possible after starting recording. Following determination of resting potential, for all other measurements the pre‐stimulus membrane potential was set to −80 mV (this value includes the junction potential correction—the amplifier reads −65 mV during the experiment) using the appropriate amount of current injection (x =, Fig. 1). Membrane resistance was analyzed in multiple ways. The first measure (*R*_in‐exp_) assessed resting input resistance independently of the “sag”‐producing *I*_h_ activation that occurs during hyperpolarizing current steps. This was calculated using Ohm's law from the amplitude of an infinite time extrapolation of a single exponential curve fitted to the membrane charging response generated by a −100 pA current injection (Fig. 1, arrow B). The exponential fit was made between points at 10 and 95% of peak amplitude. This fit was also used to determine membrane time constant and to determine one measure of the extent of sag (see below). Secondly, the reduced measure of input resistance, which includes the contribution from additional *I*_h_ activation (*R*_in‐ss_) was calculated using Ohm's law by determining the steady‐state (post‐sag) voltage deflection produced by a 500 ms, −100 pA stimulus (Fig. 1, arrow C). Finally, the input resistance at −80 mV (*R*_in‐slope_) was also measured (as the reciprocal of slope conductance) using linear regression of the steady state voltage responses elicited by a series of eight low amplitude (−50 to +30 pA), 500 ms duration current steps (Fig. 3A).

Hyperpolarization‐activated sag was measured in two ways. The first measurement (sag_sub_) simply expressed the difference between the peak (Fig. 1, arrow A) and steady state hyperpolarizations produced by a 500‐ms, −100‐pA current injection as a percentage of the peak hyperpolarization, i.e., 100 * (A–C)/A (in Fig. 1). The second measure of sag (sag_fit_) measured the decay in response relative to the amplitude of the infinite time extrapolation used to determine R_in‐exp_ i.e., 100* (B–C)/B (Fig. 1). In addition to sag, the amplitude of the sag‐related rebound depolarization was also measured relative to the pre‐stimulus membrane potential (Fig. 1, arrow D). All the passive properties were statistically compared between groups using unpaired Student's *t*‐test.

Membrane resonance was measured with standard “ZAP” protocols, in which a low amplitude sinusoidal current injection of linearly increasing frequency was applied to the cell causing voltage deflections of around 5–10 mV, as previously described (Hu et al., 2002). The impedance profile was calculated as the ratio between the fast Fourier transform of the voltage response (V(fft)) and current injection (I(fft)): *Z* = V(fft)/I(fft); the impedance versus frequency profile was subsequently smoothed with a moving average function with a span of 35 data‐points. The parameters analyzed to test the effect of treatment on the sub‐threshold resonance properties of CA1 pyramidal neurons were the peak frequency, the peak impedance and the quality factor of the resonator Q; unpaired Student's *t*‐test was used to test the effect of treatment on resonance.

Depolarizing current injections of 500 ms duration and amplitude varying stepwise from 50 to 300 pA were used to elicit action potential (AP) firing. From these, the relationship between the stimulus amplitude and the number and pattern of APs elicited was examined. The firing properties illustrated in Figures 4 and 5 were examined between groups using two‐way analysis of variance (ANOVA). To assess individual AP waveforms, the first spike fired by a 300 pA current injection was analyzed, as this size of stimulus elicited an AP in all cells. AP threshold was determined from phase plots as the voltage, at which dV/dt surpassed 20 V s^−1^ (Naundorf et al., 2006). Spike width was measured at −15 mV, which is approximately halfway between threshold (∼ −60 mV) and action potential peak (∼ +30 mV). The AP properties were statistically compared between groups using unpaired Student's *t*‐test.

Voltage responses were also measured to single, brief (2 ms), strong (2 nA) current injections. This stimulus usually elicits a single AP, which is followed in hippocampal pyramidal cells by a fast ADP, followed in turn by a brief small after‐hyperpolarization (AHP). Unpaired Student's *t*‐test was used to compare the ADP and AHP elicited by a single spike.

Finally, to further test the effect of treatment on post‐burst firing AHP, trains of 5–10–15–20–25 2 ms/2 nA square current pulses were delivered at a frequency of 50 Hz; the medium AHP was measured as the most hyperpolarized point in the 500 ms following the descending phase of the last AP reaching 0 mV. Two way ANOVA was used to statistically assess the effect of treatment on the medium AHP amplitude elicited by 50 Hz spike trains.

## RESULTS

Our studies of the effects of exogenous Aβ on IE of CA1‐PC were focused on slices treated with 500 n*M* peptide (initial monomer concentration). Although high compared to global average levels in AD brain measured in Aβ‐overexpressing transgenic mouse models of amyloidopathy (∼0.5 n*M*) (Waters, 2010), this concentration is probably the most commonly used in neurophysiological studies of brain slices, including a large body of data on Aβ effects on synaptic plasticity (Lambert et al., 1998; Lauren et al., 2009; Ma et al., 2011; Parameshwaran et al., 2007; Puzzo et al., 2005; Randall et al., 2010; Wang et al., 2004). The concentration of Aβ 1–42 soluble oligomers tends to decrease with age; this is particularly true in AD patients, where the ratio between the insoluble:soluble fraction of Aβ is significantly higher, as showed in >60 yrs post‐mortem human AD brains versus age‐matched controls, presumably because of the aggregation of soluble Aβ into insoluble fibrils and plaques (van Helmond et al., 2010). The physiological relevance of using this concentration (500 n*M*) relies on the fact that the average levels of Aβ 1–42 soluble oligomers in the AD brain can disguise the peri‐plaque concentration gradient of this species, which might differentially affect the neuronal function depending on the distance from the plaque itself.

**Figure 2:**
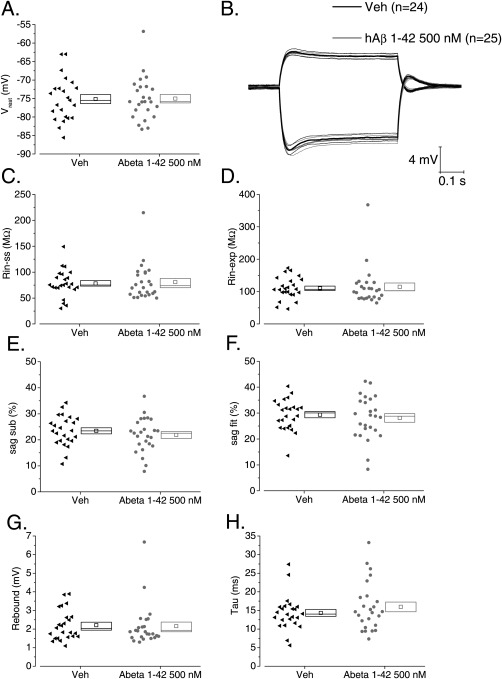
Pretreatment with soluble hAβ 1–42 oligomers does not alter sub‐threshold intrinsic properties in CA1‐PC (A) A scatter/box plot of zero current potential recorded from vehicle and Aβ pre‐treated CA1‐PC. In this and all other similar plots, the symbols to the left represent data from individual neurones, whereas the box to the right plots the mean (central symbol) plus the upper and lower bounds of the standard error and the median. In this and all other figures, data from vehicle treated neurones are presented in black and data from Aβ pre‐treated cells are shown in grey. (B) A plot of the average voltage response to both −100 (downwards) and +50 pA (upwards) current stimuli applied to CA1‐PC. The thicker central line corresponds to the mean whereas the two adjacent thinner lines represent the bounds encompassed by one standard error of the mean. (C–H) Scatter plots of sub‐threshold intrinsic properties derived from −100 pA stimuli applied at a fixed membrane potential of −80 mV.

**Figure 3:**
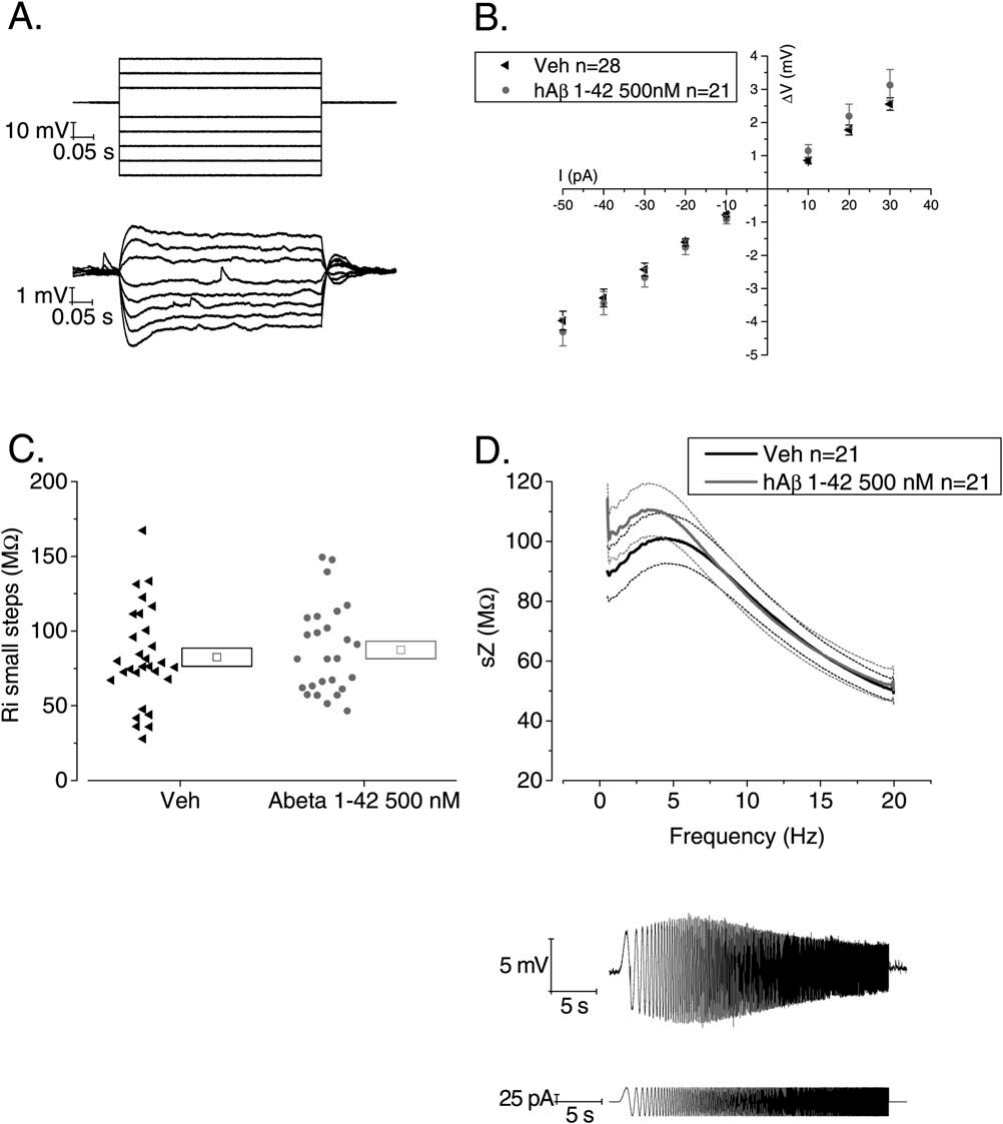
Soluble hAβ 1–42 oligomer pre‐treatment does not alter membrane resistance and impedance in CA1‐PC (A) Voltage responses (bottom) from an example CA1‐PC elicited by a series of 500 ms current stimuli varying in amplitude between −50 and +30 pA (top). (B) Pooled data from a number of recordings similar to and including the one shown in (A). The graph plots steady‐state voltage deflection versus current stimulus: the slope of the interpolated straight line for each group represents the input resistance, in accordance with the Ohm's law *V* = RI. (C) A scatter plot of input resistance derived from recordings like that in (A). Each symbol represents the slope‐derived input resistance derived from a straight line fit through all the data points obtained from a single recording. (D) The top panel shows a plot of mean impedance versus stimulus frequency for vehicle control (black) and hAβ 1–42 500 n*M*‐treated (grey) CA1‐PC. The thicker central line represents the mean values, and the dashed lines the bounds of one SEM. The bottom panel shows an example trace of the *V*_m_ of CA1 pyramidal cell resonating in response of the injection of a sinusoidal current injection of increasing frequency. The impedance Z(Ω) is measured as *Z* = V(fft)/I(fft). The quality factor of the resonator, *Q*, is calculated as the ratio between the Z at peak frequency and Z at the frequency of 1 Hz (*Q* = Z_peak_/Z_1Hz_).

Pre‐exposure for 2–5 h duration to 500 n*M* of Aβ had no effect on resting membrane potential (Fig. 2A). Figure 2B presents average voltage responses to −100 pA and +50 pA current stimuli applied at a fixed prestimulus potential of −80 mV both for vehicle and Aβ treated cells. It is readily apparent that the responses are similar. This is confirmed by the cell by cell analysis of responses to −100 pA current stimuli, presented in Figures 2C–H. Thus, membrane resistance measured as both *R*_in‐ss_ and *R*_in‐exp_ (see methods) was unaltered (Figs. 2C,D; respectively). The sag observed in response to a −100 pA hyperpolarizing current step was not altered. This was the case when the sag was measured as peak hyperpolarization relative to the steady state (sag_sub_, Fig. 2E) or using an exponential fit to the membrane charging (sag_fit_, Fig. 2F); likewise the sag‐related rebound depolarization observed on cessation of the current injection was also not altered following incubation in Aβ (Fig. 2G). Membrane time constant was also not changed by Aβ application (Fig. 2H). The lack of effect of Aβ on membrane resistance was also confirmed when an incremental series of small current pulses was applied to the cells and voltage versus current plots were constructed (Figs. 3A–C). For mean values see Table 1.

**Figure 4:**
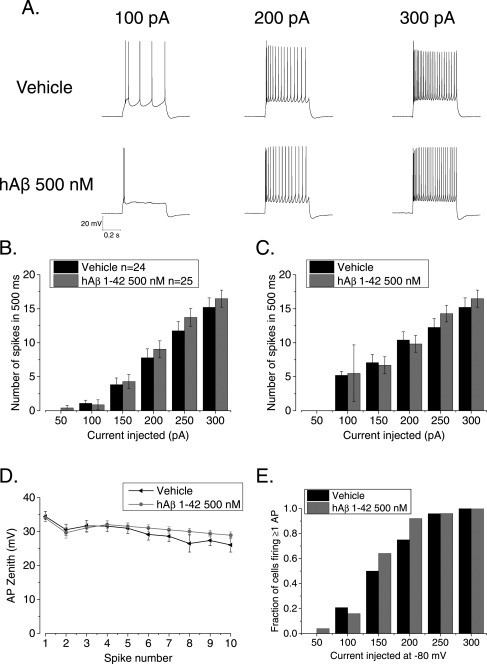
Pre‐treatment with soluble hAβ 1–42 oligomers does not alter the firing properties of CA1‐PC (A) Example traces from either vehicle or Aβ treated CA1‐PC firing APs in response of supra‐threshold 500 ms current stimuli. (B) The treatment with Aβ oligomers did not have a significant effect on the overall number of APs fired by CA1‐PC in response to 50–300 pA, 500 ms current stimuli, including or excluding (C) the cells that did not fire at least one action potential. (D) Pretreatment with Aβ oligomers did not have a significant effect on AP zenith measured within the first 10 spikes. (E) The treatment with Aβ oligomers did not alter the fraction of firing cells observed at each applied current intensity.

**Figure 5:**
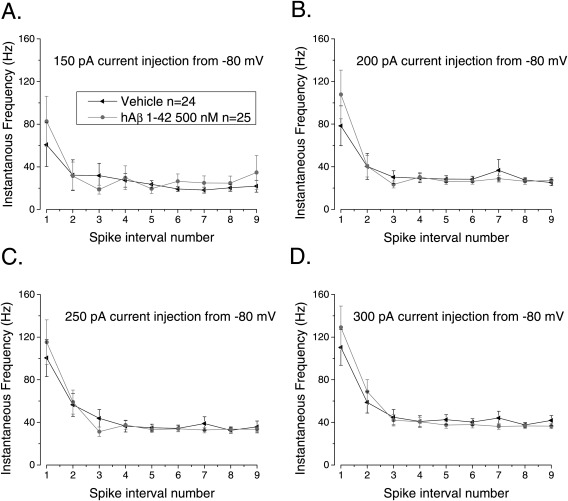
Pretreatment with soluble hAβ 1–42 oligomers does not alter the firing dynamics of CA1‐PC The instantaneous frequency of AP firing in CA1‐PC is higher during the early part of a depolarizing stimulus and subsequently accommodates to a steady slow rate of firing. Treatment with Aβ oligomers did not alter the “burstiness” of CA1‐PCs (A–D).

**Table 1:** Comparison of Passive Membrane Properties of CA1 Pyramidal Neurons Pretreated With Vehicle (DMSO = 0.05%) Versus hAβ 1–42 Oligomers 500 nM

Property	Veh. (*n* = 24)		hAβ 1–42 (*n* = 25)		*P*
	Average	SEM	Average	SEM	
RMP (mV)	−73.76	1.32	−75.04	1.15	0.47
R_in‐ss_ (MΩ)	78.36	5.36	80.88	7.03	0.78
R_in‐exp_ (MΩ)	110.28	6.89	114.68	12.03	0.75
sag_sub_ (%)	23.47	1.18	21.85	1.31	0.36
sag_fit_ (%)	29.36	1.20	28.13	1.68	0.56
Rebound (mV)	2.20	0.16	2.16	0.23	0.87
tau (ms)	14.34	0.94	15.96	1.30	0.32

The activation/deactivation properties of various ion channels with significant voltage‐dependent gating near to resting potential can create membrane resonance in neurones. In CA1‐PC this is typically seen around 5 Hz and results from the activity of HCN channels and low threshold K^+^ channels such a Kv7 family members. We analyzed resonance properties in 21 Vehicle and 21 hAβ 1–42 oligomers treated neurones. As shown in Figure 3D there was no difference between the groups. This was the case for the peak resonant frequency, the peak impedance and the quality factor *Q* of the resonator (see Table 2).

**Table 2:** Comparison of Resonance Properties of CA1 Pyramidal Neurons Pretreated With Vehicle (DMSO = 0.05%) Versus hAβ 1–42 Oligomers 500 nM

	Veh (n = 21)		hAβ 1–42 (n = 21)		*P*
	Mean	SEM	Mean	SEM	
Peak frequency (Hz)	5.17	0.43	4.58	0.51	0.38
Q	1.21	0.03	1.15	0.02	0.18
Peak Z (MΩ)	105.83	9.31	115.99	8.79	0.43

The data in Figures 2, 3 indicate we were unable to find any significant difference in sub‐threshold membrane properties between control cells and those exposed to 500 n*M* Aβ for between 2 and 5 h. This lack of alteration to sub‐threshold intrinsic properties is in good agreement with our work in transgenic mice where the major differences are seen in supra‐threshold parameters. Thus, to study AP firing patterns of CA1‐PC, we depolarized the cells with 500 ms current injections ranging in amplitude from 50 to 300 pA (Fig. 4A shows the response of an example cell from the two treatment groups at three different current intensities). The plot in Figure 4B presents the mean number of spikes fired for each stimulus amplitude (including sweeps where no spikes occurred). This illustrates that almost identical numbers of spikes were produced in the 25 hAβ 1–42 oligomers‐ and 24 vehicle‐treated cells studied. A similar outcome is apparent from Figure 4C, which also plots the number of spikes fired for each stimulus, but only includes data from sweeps in which at least one action potential was produced. For the 300 pA current injection the mean peak amplitude of the first 10 APs fired is shown in Figure 4D: no difference was observed between the two treatments. Finally, no difference was observed in the fraction of cells firing one or more APs in response to a depolarizing current step of increasing intensity (Fig. 4E).

In aged PSAPP transgenic mice stimulated with relatively weak supra threshold current injections, the pattern of AP firing in CA1‐PC during 500 ms depolarizations was significantly altered compared to wild‐type littermates (Brown et al., 2011). In particular, the transgenic mice exhibited a more pronounced burstiness soon after the onset of the current injection. We have recently seen a somewhat similar phenotype in the PDAPP transgenic line (Kerrigan et al., 2013). Such an effect is best illustrated in plots of instantaneous spiking frequency versus spike interval number. Such plots, comparing hAβ 1–42 oligomers‐ and vehicle‐treated slices for four different levels of current injection, are shown in Figures 5A–D. It is clear that a short exposure to a high (500 n*M*) concentration of hAβ 1–42 oligomers, was not able to alter the AP by AP profile of instantaneous frequency in a way similar to that we reported in the Aβ overproducing transgenic mice (Brown et al., 2011; Kerrigan et al., 2013).

The post‐spike ADP is a major determinant of burstiness in hippocampal pyramidal cells, and was larger in PSAPP mice (Brown et al., 2011). In this study, commensurate with the lack of change in AP firing dynamics, the ADP amplitude following a single spike was not modified in hAβ 1–42 oligomer‐treated slices (vehicle 16.6 ± 1.2 mV, versus hAβ 1–42 oligomer 15.2 ± 1.1 mV, *P* = 0.4). Thus, an acute 2–5 h treatment with 500 n*M* hAβ 1–42 oligomers was not sufficient to produce the same changes in IE and AP firing previously observed in Aβ‐overproducing transgenic mice.

**Figure 6:**
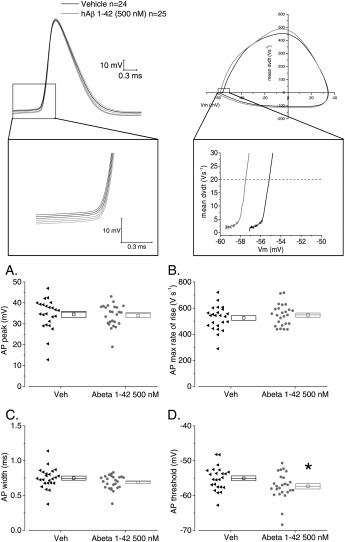
Soluble hAβ 1–42 oligomer pre‐treatment results in the hyperpolarization of action potential threshold. (Top left) The average waveforms ± SEM of the first action potential fired in response of a 500 ms duration 300 pA amplitude current stimulus for vehicle (black) or Aβ oligomers treated (grey) cells; note the more hyperpolarized AP threshold in the latter group. (Top right) A phase–plane plot of mean AP RoR vs. *V*_m_ for the two treatment groups. A‐D Scatter/box plots describing the effect of Aβ treatment on the single AP properties: only AP threshold is significantly altered, *= *P* < 0.05.

In CA1‐PC of PSAPP transgenic mice, other than finding more “bursty” firing patterns, we also identified changes to the properties of individual action potentials (Brown et al., 2011). For example, analysis of the first AP fired by 300 pA depolarizing current injections showed that, on average, APs rose more slowly, had a lower zenith, and were also narrower in width at −15 mV. The former two changes are likely to result from the deficit in Na^+^ channels we described in CA1‐PC using macropatch recording (Brown et al., 2011). In this light, we examined if acute hAβ 1–42 oligomers treatment was able to modify the AP waveform in CA1‐PC. To do this, we examined the properties of the first AP fired in response to a 300 pA stimulus (as we had performed in our work in transgenic mice). The amplitude of the AP was not modified (Fig. 6A, and see also the first data point in Fig. 4D), nor was the AP maximum rate of rise (RoR) (Fig. 6B). Furthermore, the spike width was not different when measured at −15 mV (Fig. 6C) or at spike threshold (vehicle, 1.59 + 0.09 ms versus hAβ 1–42 oligomer‐treated 1.66 ± 0.12 ms, not shown). The spike threshold, however, was altered, having moved some 3 mV more negative in the hAβ 1–42 oligomer‐treated neurones (Fig. 6D). Given that resting potentials were similar in both groups (Fig. 2A) this means that CA1‐PC in hAβ 1–42 oligomer‐treated slices require around 15% less depolarizing drive to reach spike threshold (see Table 3 for averages and SEM).

**Table 3:** Comparison of AP Properties in Control CA1 Pyramidal Neurons Pre‐treated With Vehicle (DMSO=0.05%) Versus hAβ 1–42 Oligomers 500 nM

	Veh (*n* = 24)		hAβ 1–42 (*n* = 25)		*P*
	Mean	SEM	Mean	SEM	
AP_peak_ (mV)	34.46	1.50	33.95	1.05	0.78
AP_width_ (ms)	0.75	0.03	0.69	0.02	0.10
AP_thres_ (mV)	−54.98	0.70	−57.40	0.79	0.03
AP_max_dvdt_ (Vs^−1^)	530.42	18.85	549	16.09	0.45

Following short bursts of action potential firing, hippocampal pyramidal cells exhibit an AHP. The size of this after‐potential is reported to change during normal aging (Oh et al., 2010), an effect that has been postulated to play a role in age‐related cognitive decline (Luebke and Amatrudo, 2012; Oh et al., 2010). Previous reports suggest that this AHP is modified in Aβ‐overproducing mice, although both increases (Kaczorowski et al., 2011) and decreases have been reported (Kerrigan et al., 2013).

**Figure 7:**
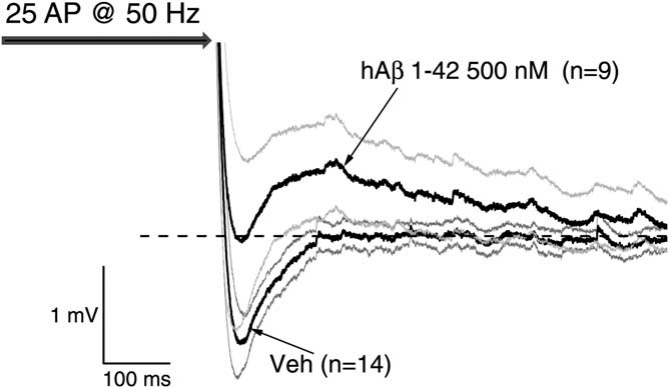
Soluble hAβ 1–42 oligomer pretreatment impairs postburst AHP. Group average post AP‐train responses from 9 Aβ‐treated and 14 control cells. A total of 25 AP were evoked in each cell as shown by the arrow. The poststimulus mean voltage responses are shown by the thick black traces and the extent of the standard error of the mean is represented by the grey lines. The dashed horizontal line represents the resting potential before the AP train (i.e., −80 mV); thus afterpotentials below this are AHPs and afterpotentials above it are ADPs. Note the presence of a clear AHP lasting around 150 ms in the control cells whereas Aβ treated cells are characterized by an ADP.

To investigate how Aβ affected AHPs following bursts of AP firing we used repeated brief, strong current injections (2 nA, 2 ms) to induce CA1‐PCs to fire trains of AP at 50 Hz. The trains consisted of 5, 10, 15, 20, or 25 APs. In CA1‐PC from control slices such stimuli reliably evoked a mAHP; in contrast, mAHPs were completely absent in many cells from Aβ treated slices. Furthermore, in the peptide treated cells a postburst ADP not seen in control cells and lasting a few hundred ms was apparent. This is illustrated in Figure 7, which presents the group average voltage responses following 25 AP evoked at 50 Hz compiled from 9 Aβ‐treated and 14 control CA1‐PC. An essentially similar finding was made for 50 Hz AP trains comprised of 5, 10, 15, or 20 stimuli. The maximal mAHP amplitude seen following each of the 50 Hz trains was measured in each cell and compared across groups. This showed a significant depression of the mAHP following Aβ (500 n*M*) treatment (*n* = 9) compared to vehicle treated slices (*n* = 14, *P* < 0.005). Similarly the AHP observed in CA1‐PC after a single AP (elicited with a single 2 nA current injection lasting 2 ms) was also around 50% smaller in Aβ treated slices (control: 1.2 ± 0.2 mV (*n* = 17) versus Aβ: 0.6 ± 0.1 mV, (*n* = 17), *P* < 0.02).

## DISCUSSION

On the basis of multiple lines of evidence, soluble Aβ peptides are still regarded by many as key pathological species in AD, potentially contributing to both pathogenesis and disease progression (Hardy and Selkoe, 2002; Hardy and Higgins, 1992). Thus, in attempts to better understand the patho‐physiological mechanisms underlying AD, numerous studies focusing on the biological actions of acutely administered exogenous soluble Aβ peptides have been made both *in vivo* (An et al., 2013; Brouillette et al., 2012; Klyubin et al., 2013) and *in vitro* (Lambert et al., 1998; Lauren et al., 2009; Li et al., 2009; Minkeviciene et al., 2009; Parameshwaran et al., 2007; Puzzo et al., 2005; Wang et al., 2004). The other major approach to understanding Aβ biology focuses on the use of mouse lines transgenically engineered to overproduce Aβ peptide species, a model of more chronic exposure to Aβ species.

Some recent studies, including our own have used Aβ‐overproducing mice to identify changes to the intrinsic electrical properties of neurones (Brown et al., 2011; Hazra et al., 2013; Hoxha et al., 2012; Kaczorowski et al., 2011; Kerrigan et al., 2013; Randall et al., 2012; Wykes et al., 2012). Due to the nature of these models, when studied experimentally brain tissues might have been exposed to increased Aβ levels, *in vivo*, for up to ∼2 years, a time only limited by the natural lifespan of laboratory mice. In this study, we investigated the effect on IE of CA1‐PC of acute *in vitro* exposure to soluble hAβ 1–42 oligomers. Clearly, this is a different scenario to chronic *in vivo* exposure, but one that has been frequently used by others with interests in determining the multifaceted functional actions of Aβ peptides.

Our investigations were based on hAβ pre‐treatment of acutely prepared mouse hippocampal slices for a period of 2–5 h. This time was selected as a balance between allowing a reasonable time for Aβ effects to be produced and consideration of the inevitable decline in the health of brain slices that occurs with time *in vitro*. Slices were treated with either soluble 1–42 oligomers 500 n*M* (which included 0.05% DMSO) or vehicle alone (i.e., DMSO 0.05%). It appears especially important to perform the correct vehicle control in studies such as this as we have recently demonstrated that 2–5 h treatment with 0.05% DMSO alters IE properties of both CA1‐PC and pyramidal neurons in perirhinal cortex (Tamagnini et al., 2014). Similarly, investigations into the consequences of acute exogenous Aβ exposure have been clouded over the years by debate over the “right” peptide preparation to use. We used an Aβ preparation that was at least shown to be active against synaptic plasticity both by others (Jo et al., 2011) and also within our own group (see methods).

In previous reports by other groups, certain neurophysiological effects of Aβ peptides have been reported to be detectable within short exposure periods. For example, the inhibition of long‐term potentiation is reported to require less than 1 h of Aβ pre‐treatment and maybe as little as 10 min *in vivo* (Cullen et al., 1997, 1996); indeed this group, who are perhaps the most prolific investigators of Aβ and hippocampal synaptic plasticity, use a standard treatment time *in vivo* of 15 mins. Also with regard to the time‐course of action of exogenous Aβ it has also been reported that a single *in vivo* injection can inhibit induction of synaptic plasticity 7 days later (Klyubin et al., 2013). Our experiments used what has been perhaps the most commonly used concentration of exogenous Aβ for *in vitro* studies of brain slices, namely 500 n*M*. We found that pre‐treatment for 2–5 h with this concentration of peptide (the same treatment that will inhibit synaptic plasticity) left many basic intrinsic properties of CA1‐PC unaltered, including resting potential, input resistance and any other sub‐threshold property. Aβ treatment did, however, produce both a negative shift in action potential threshold and a depression of the substantial mAHP that usually arises after 50 Hz trains of AP firing. Indeed, in many cells the mAHP was not only lost but appeared to be replaced with a slow ADP.

A more hyperpolarized AP threshold and a depressed AHP (and appearance of an ADP) after spike firing are both actions that would typically be regarded as a net increase in excitability. For the former this is because following Aβ exposure a smaller change from resting potential would be required to induce AP production. For the latter, the changes to spike afterpotential would enable spiking to be re‐established more readily after a prior burst. Certainly, hyperexcitability in cortical and hippocampal circuits of Aβ over‐expressing mice is an increasingly reported phenomenon, and has been discussed in the framework of the increased risk of seizures found in AD suffers, most notably in early onset cases: the changes to intrinsic properties described here may contribute to this phenomenon (Brown et al., 2011; Kerrigan et al., 2013; Minkeviciene et al., 2009; Palop et al., 2007).

An interesting insight from these results is the comparison with transgenic models of amyloidopathy, where the exposure of CA1 pyramidal neurons to increased Aβ is chronic, typically lasting months or longer. Recent studies showed that PSAPP (Brown et al., 2011), PDAPP (Kerrigan et al., 2013), and CRND8 (Wykes et al., 2012) mice do not show any difference from wild‐types in the AP threshold of CA1‐PC, but they all consistently show a decreased spike width—something we have also found in Tg2576 and TAS‐TPM Aβ overproducing mice (unpublished observations). A possible explanation of this discrepancy between the chronic and the acute models of amyloidopathy might be the increased potassium channel expression, described as an early consequence in one transgenic model (Wykes et al., 2012). Such a change could act as a compensatory response to a decreased AP threshold arising from more acute exposure to Aβ. Indeed, the presence of time‐dependent compensatory changes arising from brain plasticity are a constant challenge for those working with chronic disease models.

The global Aβ concentration in the brain of Aβ overproducing transgenic mice is likely to be much lower than the 500 n*M* Aβ 1–42 concentration used in this study for acute slice pre‐treatment. Indeed, in terms of clinical AD, the commonly used Aβ concentration we used to treat slices with is high. Mean global levels of soluble Aβ 1–42 in the brains or CSF of AD sufferers are typically in the low pM range. It remains possible, however, that higher levels are present within certain CNS niches, for example in the vicinity of amyloid plaques or perhaps within synaptic clefts.

When presenting our previous research on transgenic mice that overproduce Aβ we have often been asked if we observe similar behaviors when applying soluble Aβ peptides acutely. By performing many similar measurements to those we made previously in PSAPP (Brown et al., 2011), PDAPP (Kerrigan et al., 2013), and other mice this study has partially addressed this question here. It is, however, worth considering if it would even be reasonable to expect to see similar changes in Aβ overproducing transgenic mice and following acute Aβ treatment. Although both interventions involve exposure to Aβ the nature of the exposure is different in each case. Transgenic mice engineered to overproduce Aβ start life with normal, low Aβ loads and generate increased levels over many months, although in different mouse‐lines the rate of development and extent of the amyloid pathology is different (Lithner et al., 2011). However, at the time‐points they are usually studied, the circuits under investigation have often experienced an increased Aβ load for many months. Furthermore, as indicated above, the CNS is plastic exhibiting multiple adaptive mechanisms including changes to gene expression patterns that can remodel the physiology of cells. Thus if they are perturbed acutely, for example by Aβ, the cells of the CNS are likely, over time, to reshape their physiology in multiple ways. This could likely lead to different neurophysiological outcomes compared to those produced by acute Aβ exposure over a few hours. Relating to this, AD sufferers are now thought to reflect outcomes of detrimental processes that have been established over many years, some of which may involve Aβ: in this regard, the transgenic mouse may be a better model of aspects of the pathophysiology of the AD brain during the progression phase, whereas the acute Aβ actions may tell us important information about the early biological processes that trigger subsequent adaptive and pathological changes.

It may be useful in future to investigate the cellular basis of the changes to IE that are produced by acute Aβ treatment. The altered AP threshold is likely to be a consequence of a change to voltage‐gated Na^+^ channels. This is something which could possibly be investigated with voltage‐clamp studies of nucleated macropatches, an approach we have used previously to identify Na^+^ channel changes underlying altered IE both in mouse models of amyloidopathy (Brown et al., 2011) and associated with normal aging (Randall et al., 2012). Given the prominent role of intracellular Ca^2+^ in CA1‐PC AHPs, the altered afterpotentials seen following Aβ treatment may reflect a change to activity‐dependent intracellular Ca^2+^ signalling, something that might best be examined further with cellular imaging methodologies.
